# Phototherapy

**Published:** 1899-10-07

**Authors:** 


### PHOTOTHERAPY.

A few years ago a good deal was heard about treating small-pox in the dark, or in rooms from which all the actinic rays were excluded, in the hope of being able by this means to lessen the intensity of the eruption, and to diminish the "pitting" which so commonly follows the disease. Although there was plenty of old tradition in favour of treating small-pox by red light, the method which has of late years been put in practice has a more scientific basis, being founded on the observation that the chemical rays of light are themselves capable of causing an inflammation, and on the assumption that they might therefore be capable of aggravating an inflammation, such as the eruption of small-pox, which already existed. *Per contra*, it has been thought that an influence acting so powerfully as these actinic rays have proved themselves capable of doing upon the skin ought to possess some therapeutic power if properly applied, and with the object of ascertaining to what extent they may be usefully employed in the treatment of disease, a careful investigation of the whole subject has been undertaken by Dr. Finsen, of Copenhagen, whose results in the treatment of lupus by concentrated light are now described by his principal assistant, Dr. Bie.[1] The method of treatment which has been elaborated is founded on the following data, which have been experimentally proved: (1) The bactericidal property of the chemical rays of light; (2) the power of these rays to produce an inflammation of the skin (erythema solare), which, although formerly described under the name of erythema caloricum, has been proved to be due to the chemical and not the heat rays of the sun; (3) the power of the chemical rays to penetrate deeply into the skin. All the above data have been proved in regard to ordinary sunlight, and also in regard to the arc electric light. But for therapeutic purposes the light requires to be concentrated, otherwise the time required would be too great and the penetrating effect would not be great enough. It is necessary, however, that while the blue, the violet, and especially the ultra-violet rays are concentrated as much as possible, the heat rays shall be eliminated, and for this purpose the light is passed through certain heat-absorbing fluids. Even after this is done, however, an injurious amount of heat accompanies the light rays, and to prevent this causing injury the skin is pressed upon by a plate of quartz, between which and another plate or lens of the same material water is made to circulate. Thus the skin is kept cool. But another effect is produced by the pressure so applied, for by it the part is rendered anæmic, and this is found to aid very greatly the penetration of the light, the violet and ultra-violet, *i.e.*, the most important rays, being absorbed by blood and thus prevented from penetrating tissues in which blood exists. The most marked results have been obtained in the treatment of lupus vulgaris and lupus erythematosus. Certainly, the photographs showing the conditions before and after treatment are very striking.

[1] Brit. Med. Jour., Sept. 30.

